# Policies, delivery models, and lessons learned from integrating mental health and substance abuse services into primary health care in Ethiopia

**DOI:** 10.1096/fba.2020-00145

**Published:** 2021-06-16

**Authors:** Lia Tadesse Gebremedhin, Tedla W. Giorgis, Heran Gerba

**Affiliations:** ^1^ Minister of Health Addis Ababa Ethiopia; ^2^ Advisor to the Minister Ministry of Health Addis Ababa Ethiopia; ^3^ Director‐General Ethiopian Food and Drug Administration Addis Ababa Ethiopia

**Keywords:** health care reform, mental health recovery, noncommunicable diseases, primary health care, substance‐related disorders

## Abstract

In Ethiopia, noncommunicable diseases (NCDs) represent 18.3% of premature mortality, consume 23% of the household expenditures, and cost 1.8% of the gross domestic product. Risk factors such as alcohol, khat, and cannabis use are on the rise and are correlated with a substantial portion of NCDs. Associated NCDs include depression, anxiety, hypertension, coronary heart disease, and myocardial infarction. The multi‐faceted nature of mental health and substance abuse disorders require multi‐dimensional interventions. The article draws upon participant observation and literature review to examine the policies, delivery models, and lessons learned from the Federal Ministry of Health (FMOH) experience in integrating Mental Health and Substance Abuse (MH/SA) services into primary care in Ethiopia. In 2019, FMOH developed national strategies for both NCDs and mental health to reach its population. Ethiopia integrated MH/SA services at all levels within the government sector, with an emphasis on primary health care. FMOH launched the Ethiopian Primary Health Care Clinical Guidelines, which includes the delivery of NCD services, to standardize the care given at the primary health care level. To date, the guidelines have been implemented by over 800 health centers and are expected to improve the quality of service and health outcomes. Existing primary care programs were expanded to include prevention, early detection, treatment, and rehabilitation for MH/SA. This included training and leveraging an array of health professionals, including traditional healers and those from faith‐based institutions and community‐based organizations. A total of 244 health centers completed training in the Mental Health Gap Action Programme (mhGAP). In 2020, 5,000 urban Health Extension Workers (HEWs) participated in refresher training, which includes mental health and NCDs. A similar curriculum for rural health workers is in development. Ethiopia's experience has many lessons learned about stakeholder buy‐in, roles, training, logistics, and sustainability that are transferable to other countries. Lessons include that "buy‐in" by leaders of public health care facilities requires consistent and persistent nurturing. Ensure the gradual and calibrated integration of MH/SA services so that the task‐sharing will not be viewed as "task dumping." Supervision and mentorship of the newly trained is important for the delivery of quality care and acquisition of skills.

## 1 INTRODUCTION

In Ethiopia, the rise of noncommunicable diseases (NCDs) has resulted in increases in premature mortality and government and household expenditures for health. Risk factors such as alcohol, khat, and cannabis use are on the rise and are correlated with NCDs. Substance abuse among youth is a particular concern.

The multi‐faceted nature of mental health and substance abuse (MH/SA) disorders require multi‐dimensional interventions. The Federal Ministry of Health (FMOH) developed national strategies for both NCDs and mental health.^1^ Ethiopia integrated MH/SA services at all levels within the government sector, with an emphasis on the primary health care level.

The article draws upon participant observation and literature review to examine the policies, delivery models, and lessons learned from Ethiopia's experience in integrating MH/SA services into primary care. There were many lessons learned from Ethiopia's experience about stakeholder buy‐in, roles, training, logistics, and sustainability.

## 2 BURDEN OF NCDS

A 2019 report from the World Health Organization (WHO) estimates that NCDs cost Ethiopia at least 31.3 billion birr (US$ 1.1 billion) per year, equivalent to 1.8% of the gross domestic product (GDP). Of that, 14% of the costs are for health care, and the remaining 86% are indirect expenses due to ill health, specifically 46.8% of reduced productivity at work, 34.8% from losses from premature death, 14.06% from government health‐care expenditures, and 4.3% from absenteeism.^2^

According to Ethiopia's National Health Accounts report, 68% of NCD services in Ethiopia were financed by out of pocket (OOP) expenditures from households.^3^ The government was responsible for nearly 30% of NCD expenditure, while the contribution of philanthropic donors for such services was negligible at 1%. Overall, 23% of the total OOP expenditures in Ethiopian households are due to NCDs. While renal failure accounted for 10% of all OOP expenditures, significant household spending also goes to other NCDs, such as mental disorders (6%), cancers (5%), diabetes (2%), and injuries (2%). A 2012 study based on the Ethiopia National Health survey reported 9.1% of survey respondents reported a major depressive episode.^4^

## 3 SUBSTANCE USE

The WHO report states that the main behavioral risk factors for NCDs are tobacco use, harmful use of alcohol, unhealthy diet, and physical inactivity.^2^ The joint study between FMOH and the Global Lancet Commission on Reframing NCDs and Injuries states, "while Ethiopia has a large and diverse burden of [NCD and injuries] risk factors, risk factors such as tobacco, alcohol, and khat use is on the rise and may lead to a substantial portion of NCDs."^5^

Alcohol abuse was the sixth leading cause of death and disability in Ethiopia in the Global Burden of Disease in 2009 and 2019.^6^ In a systematic review of alcohol consumption in Ethiopia, covering 42,811 participants, 23.86% reported current alcohol consumption and another 8.94% hazardous consumption.^7^ The same study found that hazardous consumption among men was much higher than women, at 11.58.% for men compared to 1.21% for women. An epidemiological study of rural adult population (ages 15 and above) also estimates alcohol dependence lasting 12 months or more at 1.5%.^8^

Khat refers to the leaves and the young shoots of the plant *Catha edulis* Forsk, a species belonging to the plant family Celastraceae.^9^ Khat leaves are chewed habitually in the south‐western part of the Arabian Peninsula and East African countries between Sudan and Madagascar, namely Djibouti, Ethiopia, Somalia, Kenya, Tanzania, and Uganda. In Ethiopia, khat has become a cash crop, and growing khat results in profits exceeding those that can be achieved from growing other crops. Khat is chewed by 15.8% of adults.^10^ According to a comprehensive study conducted in partnership between FMOH and WHO survey, men (61%) were more likely to chew khat daily than women (50.4%).^11^

The demand for cannabis is also increasing. The Ethiopia National Drug Control Master Plan, 2017–2022, estimates cannabis use disorder represents 1.5% of mental health issues. The plan reports that arrest and seizure statistics of cannabis, cocaine, heroin, and methamphetamine between 2011 and 2017 has shown significant increases.^12^ Cannabis is illegal in Ethiopia but is still locally cultivated. In 2018–2019, the Ethiopian Federal Police Commission burned over 7 hectares of cannabis farms.^13^ A study of 695 university students revealed 4.5% lifetime usage of cannabis.^14^

## 4 SUBSTANCE USE RISKS FOR CHRONIC DISEASES

Substance abuse and mental illnesses are frequently co‐occurring disorders and services. A study of 478 individuals in Amhara Region found depression, anxiety, and stress were highly prevalent among khat chewers: 27.4%, 40.6%, and 18.8%, respectively. ^15^ In a study of 352 schizophrenic patients, 70.7% who had used khat in the past three months had a lower associated quality of life.^16^ Research conducted in Hargeisa, Somaliland has shown frequent khat usage is a risk for hypertension, coronary heart disease, and myocardial infarction.^17^ Two studies conducted in Jimma, Ethiopia showed that khat chewers were ten times more likely than non‐khat chewers to develop depression,^18^ and significant association between mental distress, frequency, and duration of khat use, including the use of alcohol.^19^

## 5 POLICIES FOR MENTAL HEALTH AND SUBSTANCE USE (MH/SA)

Multisectoral strategies are needed to control the production and consumption of substances and related NCDs. Table 1 provides a summary of relevant health sector strategic plans and guidelines from FMOH and the Ethiopian Food, Medicines and Health Care Administration, and Control Authority (FMHACA) now known as Ethiopian Food and Drug Administration (EFDA).

**TABLE 1:** Government Plans and Guidelines related to MH/SA and NCDs in Ethiopia

Year Introduced	Type	Agency	Name
2010	Strategic Plan	FMOH	Health Sector Development Plan (2010/11‐2014/15)
2012	Strategic Plan	FMOH	National Mental Health Strategy (2012/13‐2015/16)
2014	Strategic Plan	FMOH	National Strategic Action Plan for Prevention and Control of Noncommunicable Diseases (2014–2016)
2015	Strategic Plan	FMOH	Health Sector Transformation Plan (2015/16‐2019/20)
2017	Strategic Plan	FMHACA	Ethiopia National Drug Control Master Plan, 2017–2022
2019	Guidelines	FMOH	Ethiopian Primary Health Center Clinical Guidelines: Care of Children 5–14 years and Adults 15 years or older in Health Centers

In 2012, the FMOH developed its first Federal National Mental Health Strategy.^20^ The 2012–2016 policy marks an important milestone in the delivery of comprehensive and integrated mental health and substance abuse (MH/SA). The Strategy was developed consistent with FMOH's Five Year Health Sector Development Plan 2010–2015.^21^ It recommended guidelines for the development of mental health policy, plan, and program by the World Health Organization WHO, which is a five‐tiered structure known as "Optimal Mix of Services Pyramid."^22^

As advocated by the WHO, the Strategy called for integrating MH/SA services in the primary health care (PHC) service delivery platform to ensure a wide equitable, accessible, and affordable services to an estimated 110 million people.^23^ The Strategy also identified substance abuse as being a highly prevalent problem in Ethiopia, particularly among the youth population.^8^

The Strategy called for the training and mobilization of an array of health professionals, including traditional healers and those from faith‐based institutions and community‐based organizations. Sharing tasks among providers with differing levels of skills and expertise was important for implementation. Table 2 shows the specific MH/SA roles, tasks, and competencies at various levels in the health system. Supervision following training was valuable for ensuring quality and consistency. Mental health specialists such as psychiatrists and psychologists were trained on the WHO Mental Health Gap Action Programme (mhGAP) curriculum to provide population‐level oversight, consultation, and supervision.^24^

**TABLE 2:** Levels and Roles for MH/SA Services Adapted from the FMOH National Mental Health Strategy (2012/13–2015/16). Used with permission

Level	Regional / national	District (Woreda)	Local (Kebele)
Function	Provide second‐line specialist mental health care	Provide overall coordination to delivery of mental health care within the district	Support local delivery of mental health care
Location	Tertiary and referral hospitals	Woreda Health Bureaus	General hospitals and primary health centers
Providers	Multidisciplinary team of psychiatrists, psychologists, psychiatric nurses, social workers, and occupational therapists	Mental health focal person/health bureau staff	Psychiatric practitioners and nurses, working with general practitioners, general nurses, and health officers
Tasks	● Expert review of complex or treatment‐refractory cases ● Treatment in accordance with evidence‐based guidelines ● Longer‐stay inpatient care ● Rehabilitation facilities ● Specialist interventions, including individual and group psychological therapies, electro‐ convulsive therapy ● Specialist substance use interventions	● Coordinate mental health training of primary health care workers within the district, including refresher training ● Ensure a continuous supply of psychotropic medication ● Oversee the supervision structure to support PHC workers to deliver mental health care ● Monitoring and evaluation of mental health care delivery	● Provide clinical support to PHC workers in a district, through direct contact, referral pathways, and consultation ● Treatment in accordance with evidence‐based guidelines ● Active role in training PHC workers in mental health care ● Active role in advocacy for mental health care at the district level / supporting the mental health coordinators ● Short‐stay inpatient psychiatry service for acute stabilization and alcohol detoxification ● Review of complex cases or those not responding to initial care
Competencies	● Diagnosis, assessment, and ongoing management of complex or treatment‐refractory cases ● Implement specialized treatment protocols	● Knowledge of National Mental Health Strategy ● Basic knowledge in mental health regarding issues and prevention of mental illness ● Basic knowledge pertaining to organizing psychotropic medication procurement, developing of budgets for mental health services, and other administrative issues	● Diagnosis and assessments ● Treatment (medication and psychotherapies)

In light of the shortage of skilled mental health professionals in Ethiopia, the Strategy also called for task shifting schemes recommended by the WHO for accelerated training and expansion of a cadre of MH/SA service providers. Moreover, as there was a shortage of specialized mental health professionals, which constituted only 0.26% of the national health workforce, the strategy also called for a substantial increase in psychiatrists, psychologists, and allied mental health professionals.^20^, ^25^

In 2014, FMOH launched its first National Strategic Action Plan for Prevention and Control of Noncommunicable Diseases (2014–2016).^26^ The plan created a national coordination mechanism for a multisectoral response to the control of NCDs and their risk factors. Primarily, the action plan focused on the delivery of essential and quality preventive and curative health services integrated across the regional, local, and district levels. The plan advocated for the conduct of robust advocacy and awareness‐raising activities targeting to reach policymakers, critical stakeholders, and the population at large.

In 2015, FMOH revised its 5 Year Health Sector Transformation Plan (2015/16‐2019/20).^27^ The plan identified NCDs as a significant public health problem and proposed NCD interventions to be integrated and implemented in the existing health infrastructure. The plan also proposed Universal Health Coverage (UHC) as a key requirement for effective prevention and control of NCDs.

The integration of MH/SA services in PHC service delivery platforms meant that there would be equitable access to care both in the urban and remote rural areas close to where people live. Historically, more than 85% of Ethiopians who live in rural areas and who needed MH/SA services had to travel on a costly venture seeking treatment to the capital city of Addis Ababa or the very few large metropolitan cities. In the urban areas, there are a handful of inundated psychiatric facilities challenged with shortages of psychotropic medications and skilled mental health practitioners.^20^

In 2020, Ethiopia collaborated with the United Nations Office on Drugs and Crime and other stakeholders reached a consensus for the development of a drug policy and strategy on demand reduction, prevention, treatment, and rehabilitation.^28^

## 6 DELIVERY MODELS FOR PRIMARY HEALTH CARE (PHC)

**FIGURE 1:**
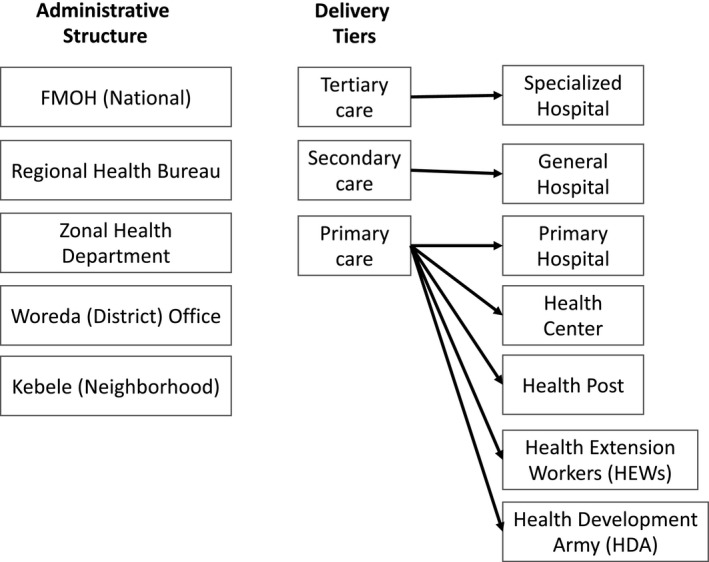
Health System Structure of Ethiopia. Adapted from the Ethiopian Health Sector Transformation Plan, 2015. Used with Permission

MH/SA services were integrated into the PHC level. The structure of the government‐run health sector in Ethiopia is shown in Figure 1. Perhaps the most renowned part of Ethiopia's health sector is the prevention and primary care offered through the Health Extension Worker (HEW) Program. ^29^

The HEW program has been celebrated by many as having been the platform that Ethiopia used to meet the health‐related Millennium Development Goals. By 2015, according to the MOH, more than 38,000 HEW have been trained and deployed in 16,440 health posts. Moreover, there were also 3,547 health centers and 311 hospitals established in the previous two decades. HEWs were formally trained to transfer knowledge and skills to families they serve so that households have better control over their health and facilitate referrals up and down the service delivery system.^30^, ^31^

The following are the innovative characteristics and unique contrast features of HEWs:

● HEW program is innovative, home‐grown, and culturally competent, rather than an imported foreign scheme imposed by outsiders.
● Committed women staff are carefully recruited and selected from their local communities, with cultural and linguistic competence and credibility, rather than persons with no relevant attachment to the local scene.
● Services are organized in a holistic manner and cover a range of priority public health issues for the broader masses, rather than piecemeal, for the selected few and controlled by external stakeholders.
● HEW program is a fully integrated and permanent part of the health system, rather than a tangential scheme at the mercy of external stakeholders’ whims, desires, and purse.
● HEWs are supported by comprehensive and robust regular career path training, rather than accidental, sporadic, and unpredictable in‐service training and temporary deployment.
● Staff are permanent on the government payroll, rather than temporarily engaged by the largess of unpredictable and unsustainable goodwill basis or foreign grants.

One study in the Lancet commented, "Ethiopia had created a model to improve PHC for others to follow."^32^ Due to a strong political commitment and country ownership of health programs, Ethiopia was able to successfully integrate and leverage MH/SA services in PHC.

Ethiopia has integrated of SA/MH into the PHC utilized the WHO mhGAP approach in four pilot sites to enable non‐mental health workers to address the basic needs of people suffering from mental, neurological, and substance use (MNS) disorders.^33^, ^34^ In 2014, in the mhGAP program, a total of 244 health centers were involved though their current functional status is not well known. However, in 2020, the mhGAP version 2.0 was adapted, and in coordination with the Regional Health Bureaus, health centers where services were deemed operational and 80 health centers from the previous round were selected for follow up training. Additionally, 400 health centers had already implemented the Primary Health Clinical Guidelines in 2018.

In 2016, FMOH, in partnership with the Johns Hopkins University and the support of the Gates Foundation, established the International Institute for Primary Health Care, intending to strengthen the internal processes and management of the HEW and PHC systems and also to contribute to the global movement Health for All.

To ensure the retention and upgrading of competencies, in 2020, 5,000 urban HEWs participated in a periodic refresher training, which includes mental health and NCDs. A similar curriculum for rural health workers is currently in development.

## 7 LESSONS LEARNED

While there have been significant successes in scaling up MH/SA services, there were also significant challenges that have to be overcome in practice. Lessons learned were gleaned and distilled from site visitation reports by FMOH staff, feedback from Regional Health Bureaus, implementing stakeholder meetings, and including mental health professionals from academic institutions who took leadership in providing training. Specifically, key lessons learned from the MH/SA policies and delivery from Ethiopia's experience include:

● NCDs require the engagement of multisectoral stakeholders. Ensure engagement at all levels: FMOH, Regional Health Bureaus, Hospitals, Health Centers, governmental and non‐governmental institutions, academia, development partners, and other critical stakeholders.
● "Buy‐in" by leaders of public health‐care facilities, especially health centers, is not a one‐time effort. Ensure the consistent and persistent nurturing of "buy‐in" by all stakeholders.
● Quick wins are essential to mobilize critical mass and build confidence. The systematic identification of resource persons, sites, programs (including leveraging adequately resourced and established programs such as HIV, TB, maternal & child health), and related opportunities enables quick wins.
● Equity is an important principle to take into consideration to ensure access to those living in remote rural areas as well as persons/population(s) officially designated as “vulnerable” by governments.
● PHC providers are inundated with the delivery of multiple services. Ensure the gradual and calibrated integration of MH/SA services so that the task‐sharing will not be viewed as "task dumping."
● Supervision and mentorship of the newly trained is important for the delivery of quality care and acquisition of skills. Ensure the provision of consistent supervision at all levels of service delivery and incorporate in staff performance appraisal.
● Logistically, ensure the availability, accessibility, and affordability of NCD pharmaceutical treatments to deliver accurate quantities and timely procurement to prevent expiration.
● Institutional funding streams are essential for the sustainability of services. Ensure robust, consistent mobilization of funding to support long‐term goals.
● As mental health, substance abuse (which is highly prone to relapses), and, generally, most NCDs are chronic diseases that require long‐term treatment and an active follow up, the provision of services should incorporate proactive and purposeful engagements utilizing case management and recovery coaching approaches.
● Develop incentive schemes to ensure retention and sustainability of trained providers.
● Closely monitor implementations to ensure accountability.
● Rapid roll‐out can lead to quick results, but deliberate, phased scaling up of integrated services leads to a deeper and sustainable impact.

## 8 CONCLUSION

Mental health and substance abuse disorders are associated with an alarming number of premature deaths, out of pocket health expenses, and reduction in quality of life. In Ethiopia, NCDs, especially MH/SA, has been addressed as part of the primary care services and training for the public health sector. This approach aligns with WHO recommendations to provide MH/SA care by integrating it into the existing PHC service delivery platform. In Ethiopia, MH/SA tasks are performed by multiple levels of health providers who receive training and periodic refresher training—ranging from health‐care extension workers with one year of training to psychiatrists who have completed medical school and residency. These tasks are covered at each level of health sector, local, district, and national. Basic MH/SA services are therefore available to the public at large, no matter where in the country they live and what type of health facility or provider is closest.

We believe the experience to date in Ethiopia will provide valuable lessons for strengthening our local efforts for equity and quality improvement for MH/SA. These lessons will also be transferrable to other countries planning to introduce or to scale MH/SA across the communities they serve. Key lessons include the importance of committed leadership by policymakers, “buy‐in” by critical stakeholders, calibrated integration of MH/SA services to enable task‐sharing across different levels health‐care providers, and provision of adequate supervision and mentorship to trainees to ensure proper acquisition of skills for delivery of quality care. Moreover, we have also found that program monitoring and the mobilization of adequate financial and human resources are essential factors to ensure quality and sustainability of MH/SA within PHC.
